# A Robust Electrochemical Aptasensor Based on a AuNP/Chitosan Conductive Network for Saxitoxin Detection in Freshwater Samples

**DOI:** 10.3390/bios16090489

**Published:** 2026-09-03

**Authors:** Luyang Zhang, Zongyu Yan, Zaiyu Zhang, Ziran Wang, Guorui Zhao

**Affiliations:** 1 Key Laboratory of High Efficiency and Clean Mechanical Manufacture, School of Mechanical Engineering, Shandong University, Ministry of Education, Jinan 250100, China; 2 State Key Laboratory of Robotics and System, Harbin Institute of Technology, Harbin 150001, China

**Keywords:** STX aptasensor, AuNP/Chitosan, saxitoxin, freshwater samples

## Abstract

Saxitoxin (STX) is a highly potent marine biotoxin, and trace contamination in aquatic environments can pose serious risks to human health. Therefore, reliable detection of low-concentration STX is crucial for water safety monitoring. Here, we developed a robust electrochemical aptasensor based on a gold nanoparticle/chitosan (AuNP/CS) conductive network for STX detection in freshwater samples. The chitosan matrix provides a three-dimensional scaffold for aptamer immobilization, while interconnected AuNPs create efficient electron-transfer pathways across the sensing interface. This integrated architecture improves interfacial conductivity and supports stable target-induced aptamer recognition. The aptasensor exhibits a linear response from 1 to 1000 nM and a limit of detection of 0.74 nM, with an apparent dissociation constant K_d_ = 70.29 ± 29.2 nM, together with high batch-to-batch consistency and long-term stability, retaining 93% of its initial response after 25 days. In spiked freshwater samples, the aptasensor achieved recoveries ranging from 99.10% to 111.33%, demonstrating the practical potential of this platform for monitoring STX contamination in aquatic environments.

## 1. Introduction

Paralytic shellfish poisoning [1,2,3,4] (PSP) is a potentially life-threatening illness caused by the consumption of seafood contaminated with saxitoxin (STX). STX [5,6], a representative and highly toxic member of this toxin family, blocks voltage-gated sodium channels and disrupts neuronal signal transmission [7]. Clinical symptoms can develop rapidly after exposure and range from gastrointestinal distress and paranesthesia to neuromuscular paralysis and respiratory failure. To protect consumers, regulatory limits have been established for STXs in seafood. For example, the US Food and Drug Administration sets an action level of 80 μg STX equivalents per 100 g (about 2.67 μmol/L) of shellfish meat [8]. Rapid and reliable STX detection is therefore essential for safeguarding food safety and public health.

Several analytical methods [9,10,11,12,13,14] have been developed for detecting STX. Although the mouse bioassay has historically been used for routine toxicity assessment, it raises ethical concerns and provides limited quantitative accuracy and toxin specificity. Instrumental methods, including high-performance liquid chromatography [15] with fluorescence detection and liquid chromatography–mass spectrometry [16,17], offer high sensitivity and congener-specific analysis. However, these techniques generally require expensive equipment, trained personnel and labor-intensive sample preparation, limiting their suitability for on-site screening [18,19]. Immunoassays and receptor-binding assays offer higher throughput but can be affected by cross-reactivity, limited reagent stability and matrix interference. Other approaches, including thin-layer chromatography, capillary electrophoresis and surface-enhanced Raman spectroscopy, have also been investigated. Nevertheless, their practical application can be constrained by insufficient sensitivity, limited reproducibility or complex instrumentation [20,21]. Portable analytical platforms that combine sensitivity, specificity, operational simplicity and robustness are therefore still needed.

Electrochemical [22,23,24,25] sensors offer a promising route towards rapid and decentralized toxin detection because of their high sensitivity, low cost and compatibility with miniaturized devices. Their analytical specificity can be further improved by integrating molecular recognition elements [26,27,28,29]. Aptamers are single-stranded DNA or RNA molecules selected through the systematic evolution of ligands by exponential enrichment (SELEX). They fold into target-dependent three-dimensional structures and bind their targets with high affinity and specificity. Compared with antibodies, aptamers generally offer greater chemical stability, straightforward synthesis and modification, lower batch-to-batch variability and potential renderability [30,31,32,33]. Aptamers functionalized with redox reporters, such as methylene blue (MB), can directly convert target-induced conformational changes into measurable electrochemical signals. Hence, these features make redox-reporter-functionalized aptamer electrochemical sensors particularly well suited for sensitive, stable and decentralized detection of STX in water samples.

The M-30f aptamer was selected as the recognition element based on previous reports. The original STX-binding aptamer, APTSTX1, was first reported by Handy [34] et al.; however, its relatively limited binding affinity restricted its analytical applicability. M-30f was subsequently obtained through rational site-directed mutagenesis and truncation of APTSTX1 and was reported to exhibit substantially improved affinity toward STX [35]. Moreover, M-30f has been used in several STX aptasensing platforms, including fluorescence, SERS, and electrochemical-based assays [36,37,38]. Therefore, M-30f was selected in this study as the STX recognition element.

Here, we developed an aptamer-based electrochemical biosensor for sensitive and selective STX detection in freshwater, consisting of a gold nanoparticle/chitosan (AuNP/CS) conductive network to immobilize an MB-labeled STX aptamer. Screen-printed carbon electrodes were modified with a AuNP/CS conductive network to achieve high batch-to-batch consistency and long-term stability. Upon STX binding, aptamer reorganization modulated electron transfer between the MB reporter and the electrode surface, generating a differential pulse voltammetry signal. The chitosan matrix served as a three-dimensional scaffold for aptamer immobilization, while the AuNPs increased the electroactive surface area and promoted interfacial electron transfer, as supported by COMSOL Multiphysics 6.3 simulations. Experiment results show that the aptasensor achieved a low detection line of 0.74 nM and long-term stability for 30 days, and its performance was validated in environmental freshwater samples. This platform offers a potential basis for portable STX monitoring in aquatic environments.

## 2. Materials and Methods

### 2.1. Materials

The STX-specific aptamer used in this study had the sequence 5′-TTTTT-TTGAGGGTCGCATCCCGTGGAAACAGGTTCATTG-3′. It was modified with a C6-linked thiol group at the 5′ terminus and methylene blue (MB) at the 3′ terminus and was designated MB-Apt. A five-thymine oligonucleotide, 5′-TTTTT-3′, bearing a C6-linked thiol group at the 5′ terminus was designated T5. Both oligonucleotides were synthesized and purified by high-performance liquid chromatography by Sangon Biotech Co., Ltd. (Shanghai, China). T5 was used as a short backfilling strand to regulate probe packing and reduce steric hindrance at the electrode surface. STX was purchased from Pribolab (Qingdao, China) and stored at 4 °C. AuNP (≤500 nm) were obtained from Macklin (Shanghai, China). Medium-viscosity chitosan (200–400 mPa s), tris (2-carboxyethyl) phosphine hydrochloride (TCEP), 6-Mercapto-1-hexanol (MCH), tris(hydroxymethyl)aminomethane (Tris), sodium chloride (NaCl), calcium chloride (CaCl_2_), iron(III) chloride (FeCl_3_), potassium ferricyanide (K_3_[Fe(CN)_6_]), potassium chloride (KCl), magnesium chloride (MgCl_2_) and acetic acid were obtained from Macklin (Shanghai, China). Solid TCEP and tris were stored at 4 °C. Unless otherwise specified, all chemicals were of analytical grade and used without further purification.

### 2.2. Experimental Procedures

#### 2.2.1. Preparation of the AuNP/Chitosan Composite and Probe Solutions

A 1 wt% AuNP/CS solution was prepared by dissolving chitosan in 2% (*v*/*v*) acetic acid under magnetic stirring at 80 °C for 1 h. The weight ratio of CS to AuNPs is 1:0.6. The mixture was magnetically stirred for 30 min and sonicated for an additional 30 min. The resulting AuNP/CS composite gel was stored at 4 °C until use.

An immobilization buffer containing 10 mM of tris, 100 mM of NaCl, 10 mM of TCEP and 20 mM of MgCl_2_ was prepared. TCEP was included to maintain the terminal thiol groups in their reduced form. MB-Apt and T5 were separately diluted in this buffer to final concentrations of 0.5 μM and 10 μM, respectively. The resulting preparations were designated the MB-Apt and T5 functionalization solutions.

#### 2.2.2. Fabrication of the STX Aptasensor

Screen-printed carbon electrodes (SPCEs) were sequentially rinsed with ethanol and ultrapure water and dried under a nitrogen stream. The AuNP/CS composite gel (10 μL) was drop-cast onto the working-electrode surface and thermally cured at 80 °C for 1 h to form the composite interface.

The MB-Apt functionalization solution (10 μL, 0.5 μM) was applied to the modified electrode and incubated at 4 °C for 4 h, allowing for MB-Apt immobilization through Au–S bond formation. The determination of T5 concentration was carried out by the current response experiments of different T5-modified sensors in Figure S4. The electrode was then incubated with the T5 functionalization solution (10 μL, 10 μM) at 4 °C for 1 h. Thiolated T5 was used as a DNA spacer/diluent to regulate the MB-Apt interface in order to regulate probe packing and reduce steric hindrance at the electrode surface [39,40,41], as supported by previous gold-based DNA sensor studies.

The electrode was subsequently incubated with 10 mM of 6-mercapto-1-hexanol (MCH) for 3 h to passivate the remaining unoccupied AuNP surface sites. After each functionalization step, the electrode was rinsed with ultrapure water to remove unbound material. The fabricated aptasensors were stored at 4 °C until use.

A total of 10 μL of an STX standard solution at 0, 1, 10, 50, 100, 250, 500 or 1000 nM was applied to the electrode surface. As the matrix solution, 1 × PBS was selected. After incubation at 4 °C for 1 h, the electrode was rinsed to remove unbound STX before electrochemical measurement.

The apparent dissociation constant (K_d_) is an important indicator of the functional binding affinity of the surface-immobilized aptamer under sensing conditions [42].

The Hill–Langmuir fitting equation is as follows:

$$I=I_{0}+\left(I_{\max}-\;I_{0}\right)\frac{C}{K_{d}+C}$$

where ***I*** represents the response variable, which is the electrochemical signal change. *C* is the concentration of STX in the assay. ***K_d_*** is the apparent dissociation constant. ***I*****_0_** is the fitted baseline response at the lowest concentration, while ***I*****_max_** is the fitted saturation response to the highest concentration.

### 2.3. Electrochemical Measurements

All electrochemical characterizations, including differential pulse voltammetry (DPV), cyclic voltammetry (CV), and electrochemical impedance spectroscopy (EIS), were performed using a CHI660E electrochemical workstation (CH Instruments, Shanghai, China). Measurements were carried out in an electrolyte containing 1 mM of K3[Fe(CN)6] and 0.1 M of KCl.

For CV analysis, a potential range of −0.2 V to 0.8 V was applied with a scan rate of 50 mV/s. EIS measurements were conducted under a bias potential of 0.175 V, with an AC amplitude of 10 mV over a frequency range of 0.1 Hz to 1 MHz. DPV parameters included a potential window of −0.2 V to 0.6 V and a scan rate of 25 mV/s.

### 2.4. Spiking Recovery Experiments

Spiking recovery tests were performed using Baotu Spring freshwater samples and Daming Lake freshwater samples as representative environmental matrices. Surface water samples (~250 mL each) were collected from Baotu Spring and the central area of Daming Lake in clean bottles, filtered to remove suspended particulates and algae, and subsequently used as matrix solutions to prepare STX standard solutions in place of 1×PBS. All spiked samples were stored at 4 °C in the dark prior to analysis for no longer than 24 h before analysis.

No STX was detected in the unspiked samples above the limit of detection. The filtered water samples were therefore used directly, without adjustment of pH or ionic strength, to prepare STX-spiked samples at 0 nM, 50 nM, and 100 nM. The resulting samples were analyzed using the fabricated aptasensor. Spike recovery was calculated as follows: Recovery (%) = Cmeasured − C_0_/C_joined_ × 100%, where Cmeasured is the measured STX concentration, C0 is not joined STX, and Cjoined is the added STX concentration.

## 3. Results

### 3.1. Detection Principle

**Figure 1:**
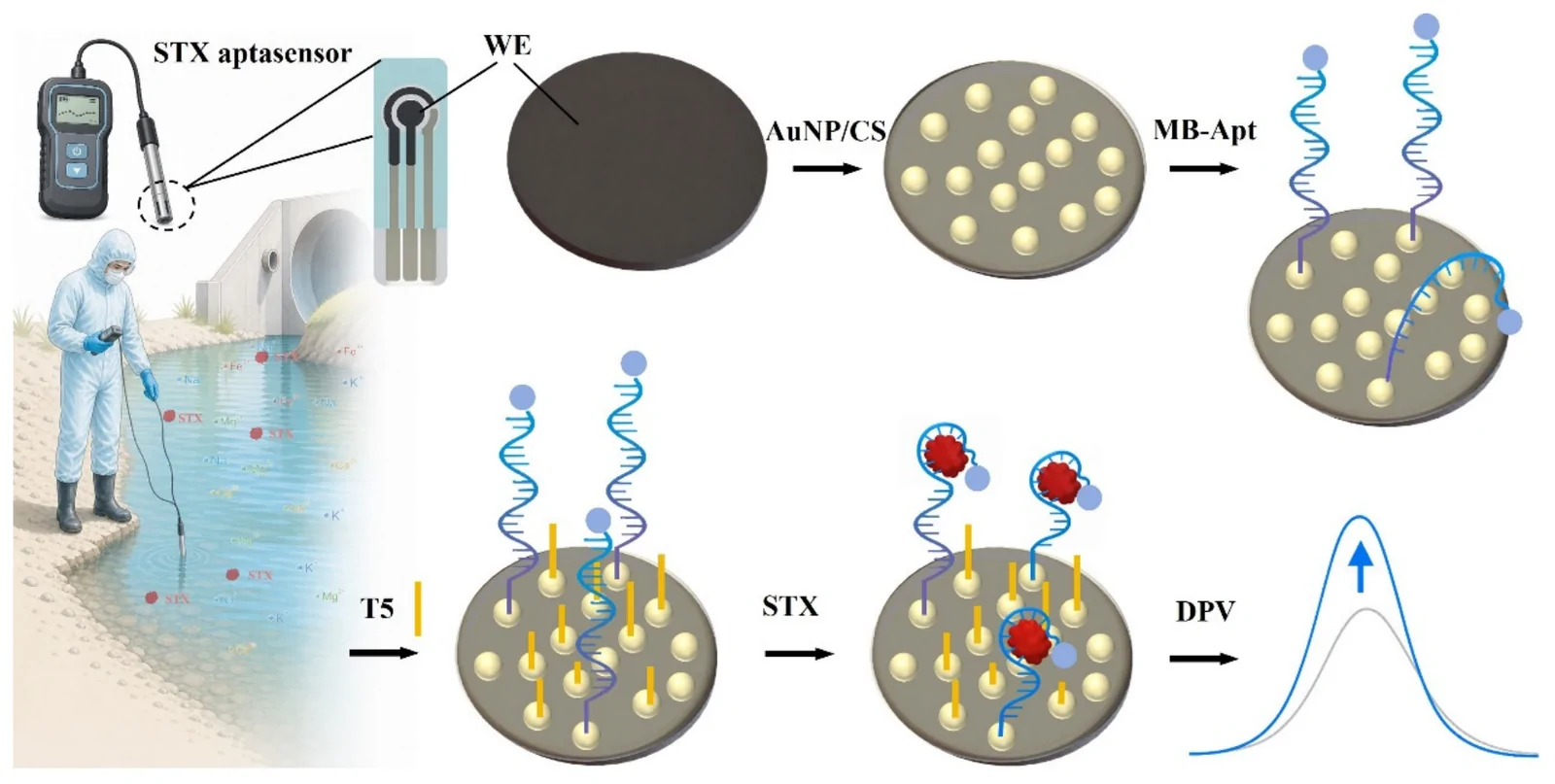
Modification process and detection principle of STX aptasensor.

The fabrication procedure and proposed sensing mechanism of the STX aptasensor are illustrated in Figure 1. The AuNP/CS composite formed on the working electrode (WE) provided a three-dimensional scaffold and conductive interface. AuNP enabled the immobilization of thiolate MB-Apt through Au–S bond formation and facilitated electron transfer between the methylene blue (MB) reporter and the WE. T5 was introduced at a T5-to-MB-Apt concentration ratio of 20:1 to backfill unoccupied binding sites on the AuNP surface. This short-strand backfilling layer was designed to regulate aptamer packing and reduce steric crowding at the electrode interface. It was therefore expected to favor a more accessible configuration of the surface-bound MB-Apt. Upon exposure to STX, the immobilized aptamer specifically bound the target. The resulting reorganization of the surface-bound aptamer was proposed to decrease the electron-transfer distance between MB and the WE. Consistent with this signal-on mechanism, the DPV peak current increased with an increasing STX concentration.

### 3.2. Simulation of AuNP/CS

Under acidic conditions, the amino groups of chitosan become protonated to form positively charged NH_3_^+^ groups. These groups interact electrostatically with the negatively charged citrate layer on the AuNP surface. Hydrogen bonding and coordination between the chitosan chains and AuNP surfaces may further stabilize the composite. As the solvent evaporates, the chitosan chains become entangled and form a continuous polymer film. The dispersed AuNPs are consequently concentrated and immobilized within the chitosan network and at the film–substrate interface. Increasing the AuNP content reduces the average interparticle distance and promotes the formation of connected conductive pathways through the chitosan matrix.

To investigate the electrical behavior of these pathways, three-dimensional representative volume element models were constructed using COMSOL Multiphysics. CS was represented as the continuous matrix, whereas each AuNP was modelled as a sphere with a radius of 25 nm. The low-AuNP model contained 24 particles and had a AuNP-to-chitosan mass ratio of 30%. The high-AuNP model contained 84 particles arranged as interconnected tortuous chains in the vertical and lateral directions, corresponding to a AuNP-to-chitosan mass ratio of 60%. These ratios refer to the relative masses of AuNP and chitosan in the simulated solid composite and differ from the experimental AuNP concentration of 0.6 wt%, which was calculated relative to the total mass of the composite solution.

**Figure 2:**
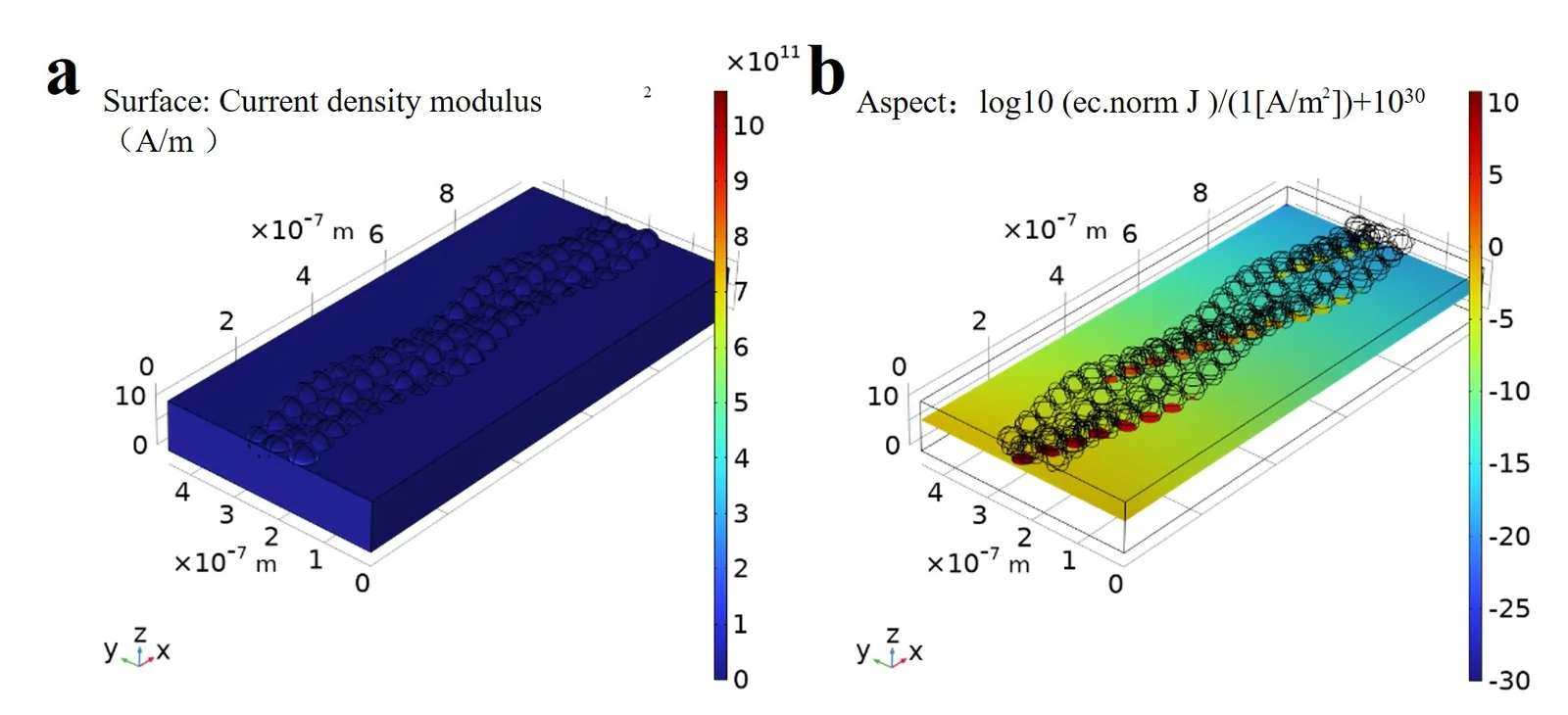
COMSOL simulation of AuNP/CS: (**a**) surface conductivity; (**b**) cross-sectional conductivity.

The electrical conductivities of AuNP and CS were set to 1.0 × 10^5^ S m^−1^ and 1.0 × 10^−2^ S m^−1^, respectively. The dimensions of the simulation domain are 1.0 μm × 0.45 μm × 0.10 μm, and the model is discretized using a Free Tetrahedral (Finer) mesh. A potential of 10 mV was applied to the left boundary, and the right boundary was grounded. All other external boundaries were electrically insulated, and current continuity was imposed at the AuNP/CS interfaces. The total current I was obtained by integrating the normal current density over the grounded boundary. The equivalent resistance was calculated as Req = ΔV/I, where ΔV is the applied potential difference across the simulation domain. In the low-AuNP model, the particles primarily formed isolated clusters, and long-range charge transport remained dependent on the chitosan matrix (Figure S1). This structure exhibited an equivalent resistance of 7.32 × 10^7^ Ω. In contrast, the high-AuNP model contained an interconnected, tortuous particle network that provided continuous conductive pathways across the simulation domain (Figure 2a,b). Its equivalent resistance decreased to 121.27 Ω, more than five orders of magnitude lower than that of the low-AuNP model. These simulations indicate that AuNP network connectivity strongly influences the effective resistance of the AuNP/CS composite and support a percolation-based explanation for the enhanced charge transport.

### 3.3. Characteristics of STX Aptasensors

**Figure 3:**
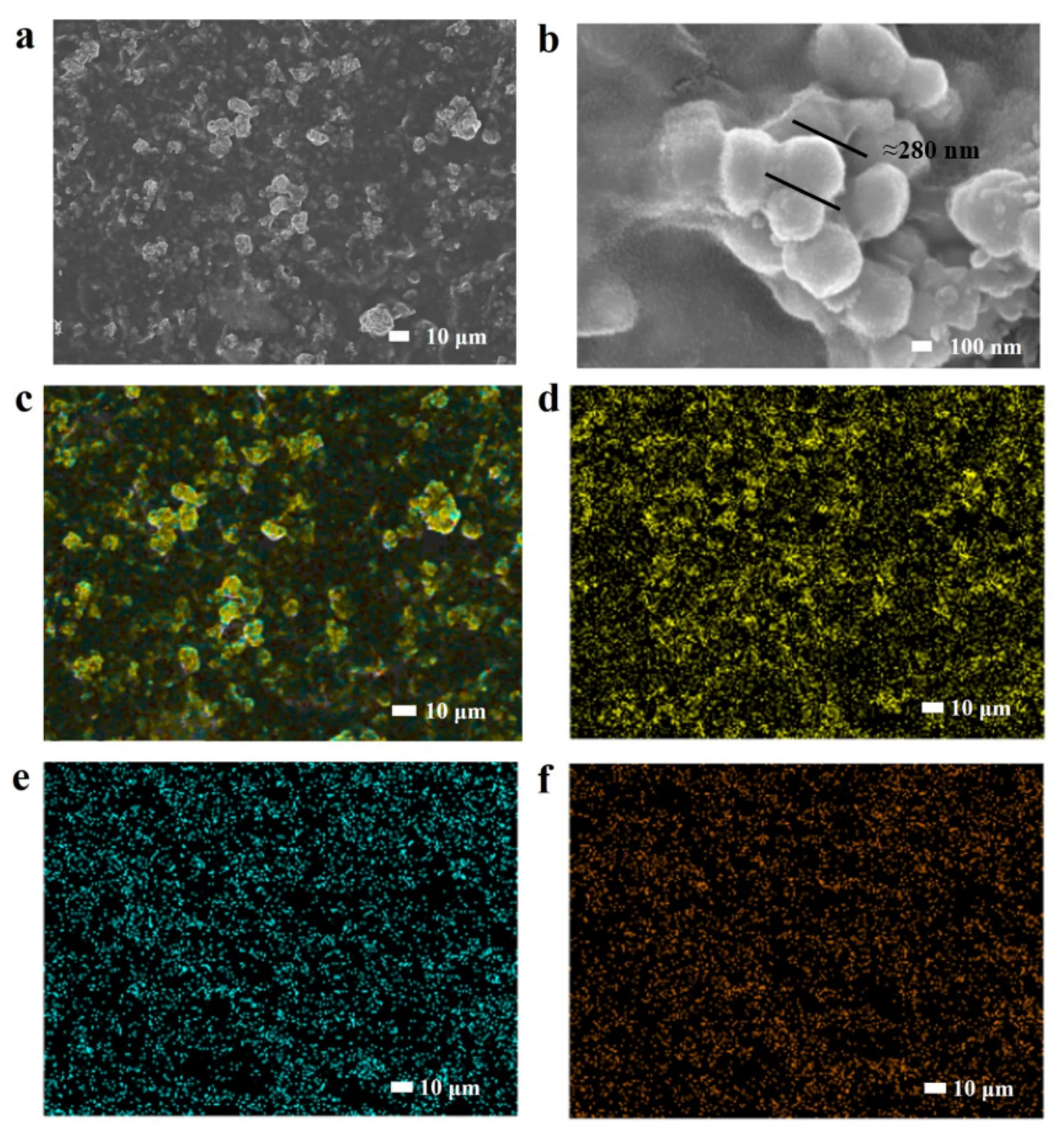
Characterization of AuNP/CS conductive network. SEM of electrode surface modified with AuNP/CS. (**a**) Scale bar, 10 µm. (**b**) Scale bar, 100 nm. (**c**) EDX distribution of Au, C, and O elements. (**d**) Distribution of Au element. (**e**) Distribution of Celement. (**f**) Distribution of Oelement.

Scanning electron microscopy (SEM) was used to characterize the morphology of the AuNP/CS composite on the working electrode (WE). The composite formed a continuous coating across the electrode surface, with AuNP broadly distributed throughout the chitosan matrix (Figure 3a). At higher magnifications, the dried chitosan matrix retained the AuNP on the electrode surface (Figure S2 and Figure 3b). The diameter of AuNPs is about 280 nm. The resulting textured morphology was expected to increase the available interfacial area, which was further evaluated by cyclic voltammetry.

Energy-dispersive X-ray spectroscopy (EDX) was used to determine the elemental composition of the modified electrode. Au, C and O were the predominant detected elements, accounting for 39.51, 26.45 and 34.04 wt%, respectively (Figure 3c). The corresponding EDX spectrum is shown in Figure 3d. Elemental mapping showed that Au, displayed in yellow, was broadly distributed across the modified electrode surface (Figure 3d). Carbon, displayed in blue, was distributed throughout the analyzed region (Figure 3e), whereas the distribution of oxygen, displayed in orange, is shown in Figure 3f. The C signal originated from both the carbon-based WE and the chitosan matrix, whereas the O signal was attributed mainly to chitosan.

**Figure 4:**
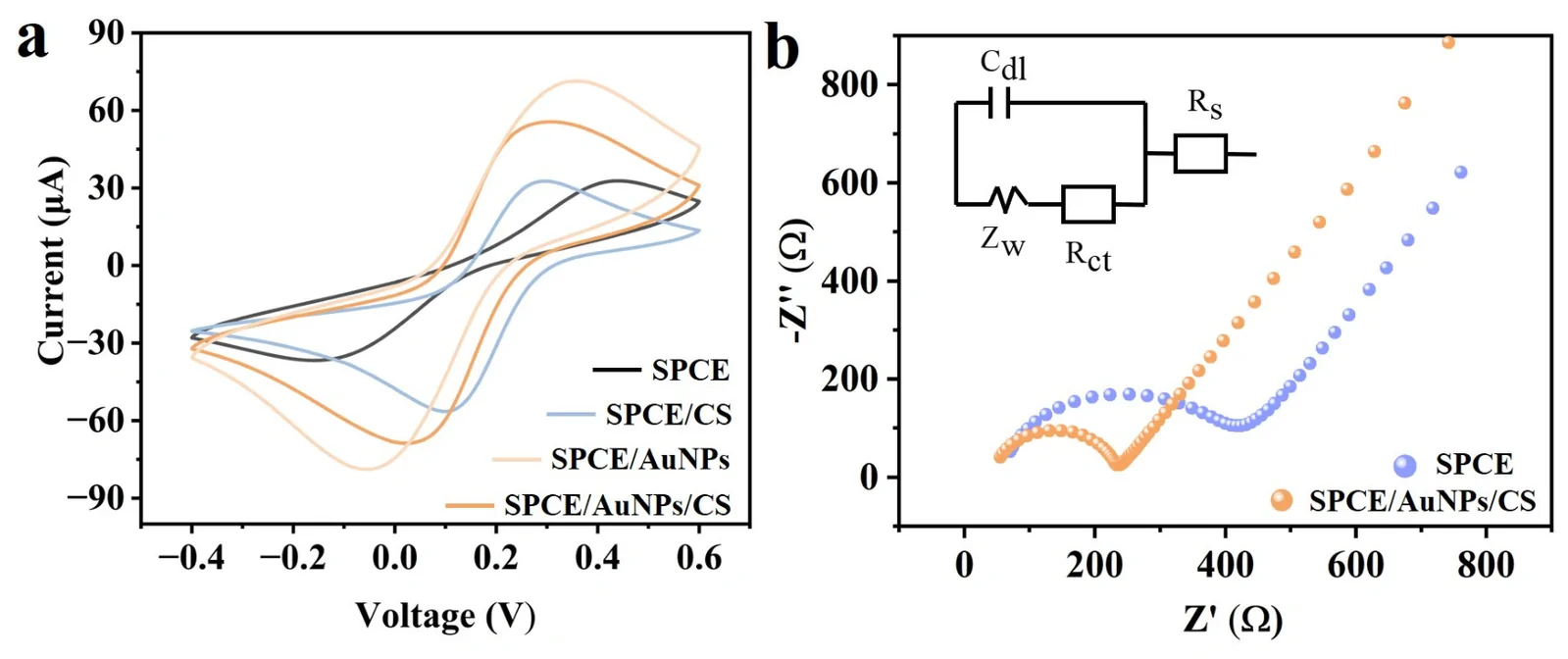
Electrochemical characteristics of AuNP/CS. (**a**) Modified AuNP/CS and unmodified CV scan curves. (**b**) AuNP/CS and unmodified EIS curves.

To evaluate whether the AuNP/CS conductive network improved electron transfer at the working electrode, cyclic voltammetry (CV) was performed using 1 mM of K_3_[Fe(CN)_6_] in 0.1 M of KCl as the redox electrolyte. CV curves of the bare SPCE and AuNP/CS-modified SPCE were recorded from −0.4 to 0.6 V at a scan rate of 50 mV s^−1^ (Figure 4a). The electrochemically active surface area (ECSA) was estimated from the cathodic peak current using the Randles–Sevcik equation: Ip = 2.69 × 10^5^ n^3/2^ A D^1/2^ C v^1/2^, where Ip is the peak current, n is the number of transferred electrons, A is the ECSA, D is the diffusion coefficient, C is the concentration of K_3_[Fe(CN)_6_], and v is the scan rate. The calculation used D = 7.6 × 10^−6^ cm^2^ s^−1^ and n = 1.

For SPCE working electrodes, the cathodic peak current was −38 μA, corresponding to an ECSA of 0.2292 cm^2^. After modification with AuNP/CS, the cathodic peak current increased to −69 μA, giving an ECSA of 0.4161 cm^2^ and a roughness factor of 2.1198. These results indicate that the AuNP/CS composite increased the electrochemically accessible area and improved the interfacial electron-transfer capacity of the electrode.

To further verify the detection performance of the AuNP/CS electrode, EIS impedance was used to test the charge transfer impedance of the electrode. The impedance of the modified AuNP/CS conductive network and unmodified electrode was detected in a 0.1 M KCl electrolyte in a 1 mM K3[Fe(CN)6] and 0.1 M KCl solution. EIS analysis (Figure 4b) shows that the Rct of the AuNP/CS conductive network is 2.35 times lower than that of the carbon electrode (from 435 Ω to 185 Ω), indicating enhanced charge transfer kinetics.

### 3.4. Performance Testing of STX Aptasensors

**Figure 5:**
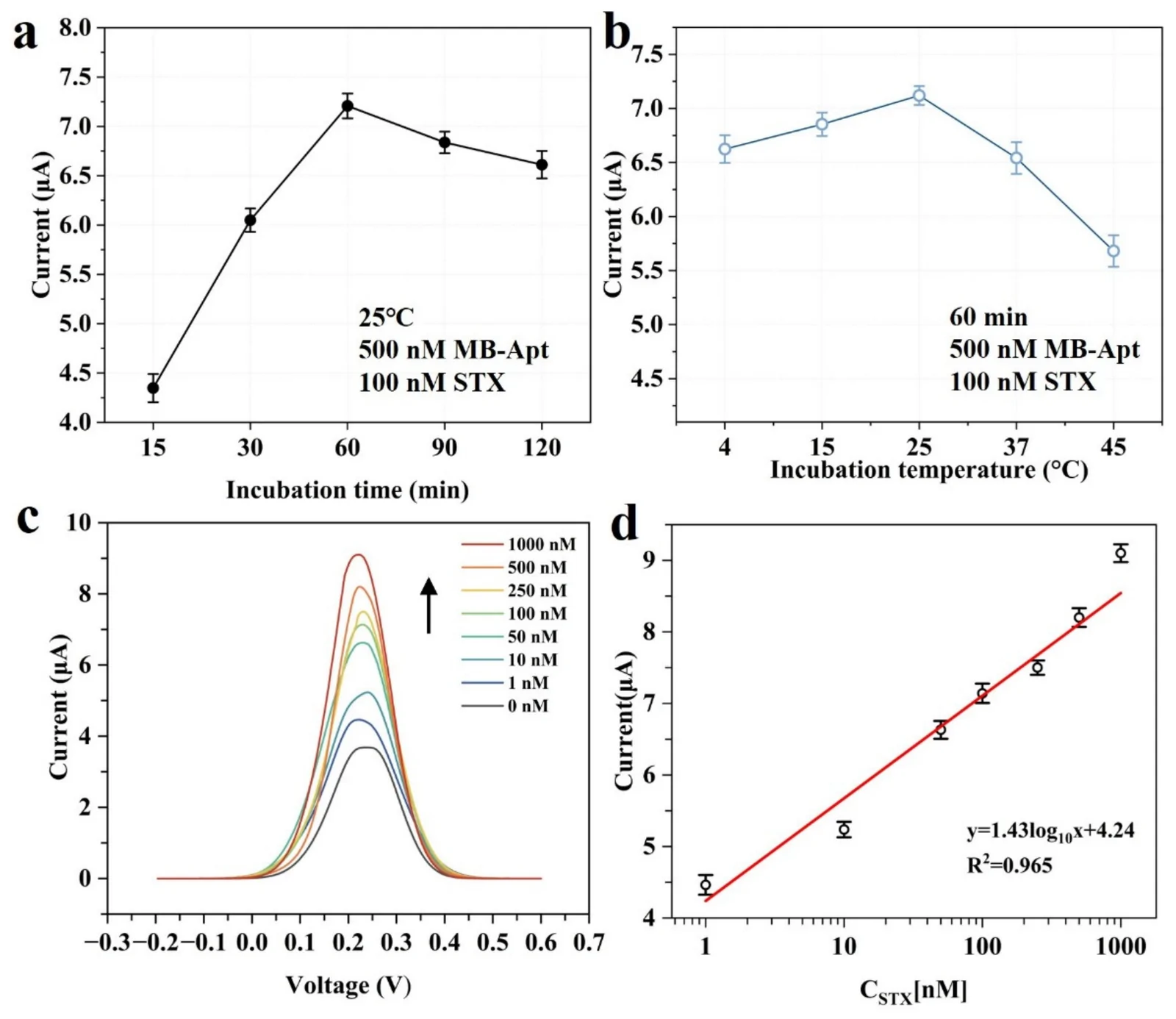
Performance evaluation of STX aptasensors. (**a**) Optimization of STX incubation time. (**b**) Optimization of STX incubation temperature. (**c**) DPV reduction peak current curve within the concentration range of 1–1000 nM. DPV responses of the MB-Apt/CS/AuNP/SPCE sensor upon incubation with STX at concentrations ranging from 0 to 1000 nM. Arrow indicates the direction of decreasing STX concentration. (**d**) Calibration curve, n = 3.

The STX aptasensors were incubated with 100 nM of STX at 25 °C for 15–120 min and analyzed by DPV (n = 3). As shown in Figure 5a, the MB reduction peak current increased from 4.35 μA (15 min) to a maximum of 7.20 μA at 60 min, indicating that toxin–aptamer binding approached equilibrium with optimal reproducibility. Prolonged incubation (90–120 min) caused a gradual current decline and enlarged error bars, likely due to partial aptamer dissociation from the AuNP surface or conformational rearrangement of the STX–aptamer complex that hindered electron transfer. Thus, 60 min was selected as the optimal incubation time to maximize signal intensity while maintaining interfacial stability.

With the incubation time fixed at 60 min, the sensor response was evaluated at 4–45 °C (n = 3). Figure 5b reveals a bell-shaped dependence: the current increased from 6.624 μA at 4 °C to a peak of 7.120 μA at 25 °C, then declined to 5.681 μA at 45 °C. The low response at 4 °C reflects sluggish diffusion and slow aptamer folding kinetics, whereas the drop above 37 °C is attributed to thermal dissociation of the toxin–aptamer complex and potential destabilization of the G-quadruplex and chitosan film. A temperature of 25 °C (room temperature) was chosen as the optimal incubation temperature, offering the highest sensitivity without external temperature control. All subsequent experiments were performed under this condition. For optimization of the STX incubation temperature, DPV current responses of the STX aptasensor were incubated with 100 nM of STX at various temperatures (4–45 °C) for 60 min. Error bars represent the standard deviation of measurements from three independent electrodes. The highest current response was obtained at 25 °C, balancing binding efficiency and interfacial stability.

The analytical performance of the STX aptasensor was evaluated by differential pulse voltammetry (DPV), based on the redox response of the methylene blue (MB) reporter on the electrode-confined aptamer. STX standards were prepared in PBS and incubated with the fabricated aptasensor before DPV measurement. In the absence of STX, the aptasensor showed a low background peak current of approximately 3.68 μA. As the STX concentration increased from 1 to 1000 nM, the MB peak current gradually increased (Figure 5c), indicating a concentration-dependent signal response.

Calibration measurements were performed with three independent replicates for each concentration (n = 3). As shown in Figure 5d, the aptasensor exhibited a strong linear relationship between peak current and STX concentration over the range of 1–1000 nM, with a correlation coefficient of R2 = 0.965. The calibration equation is expressed as y = 1.43log10x + 4.24, where y represents the DPV peak current, and x represents the STX concentration. The limit of detection (LOD), calculated using LOD = 3σ/S, where σ is the standard deviation of the blank signal, and S is the slope of the calibration curve, was 0.74 nM. According to Figure 5c,d and the Hill–Langmuir fitting equation, K_d_ = 70.29 ± 29.2 nM, was calculated. These results demonstrate that the proposed aptasensor enables sensitive electrochemical detection of STX over a broad concentration range. Figure S3 shows the binding ability of STX to the electrode surface MB-Apt, demonstrating the effectiveness of modifying MB-Apt.

**Figure 6:**
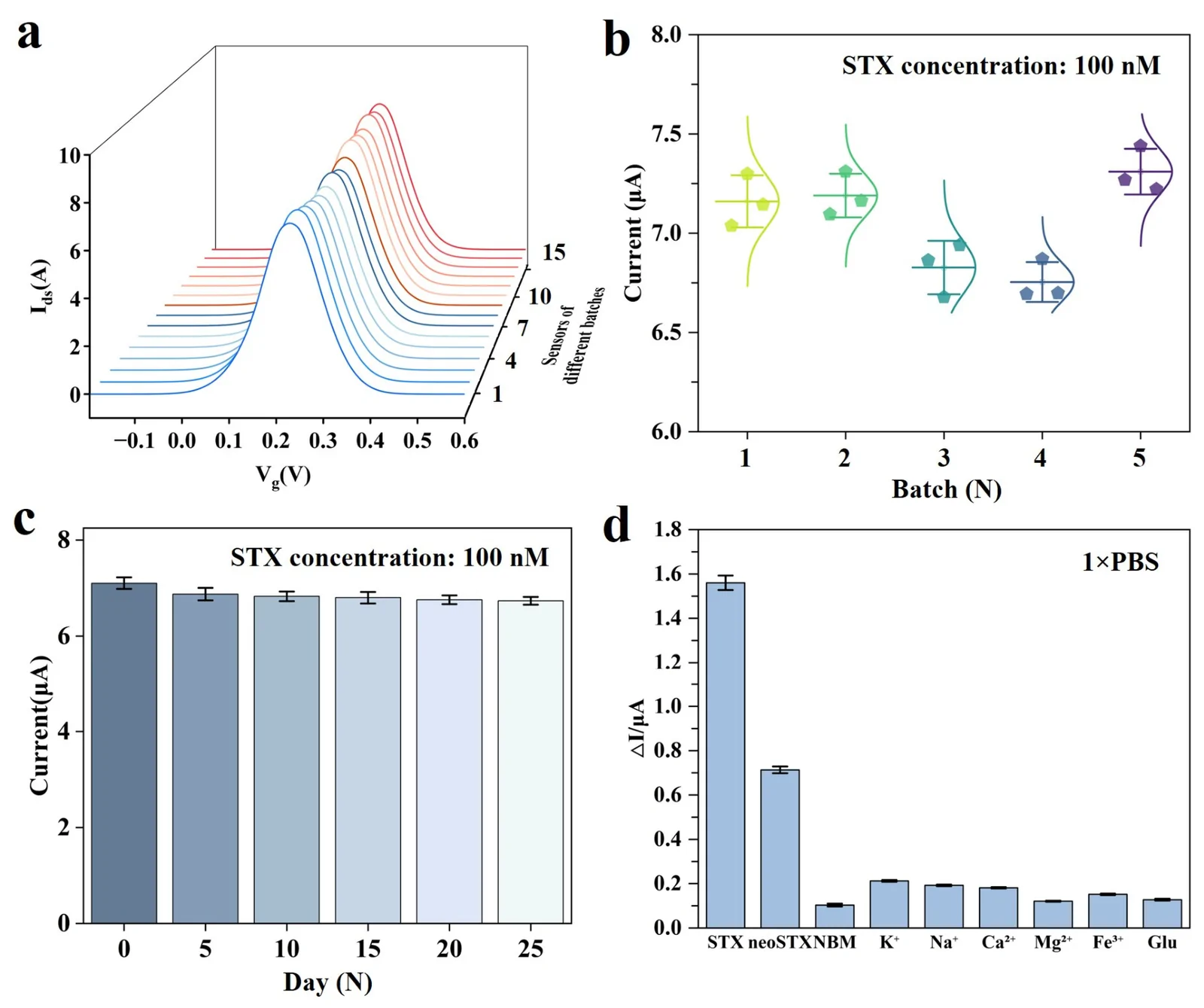
Consistency evaluation of STX aptasensor. (**a**) Under the same measurement conditions, 15 independently manufactured electrodes from 5 groups responded to a DPV current of 100 nM STX. (**b**) Statistical analysis of inter-batch consistency of current response for 5 groups of electrodes. (**c**) To determine the long-term storage stability of the sensor, it was stored in a dryer at 4 °C. (**d**) Selective adaptation of STX aptasensors. The detected substances include: STX (10 nM), neoSTX (10 nM), NBM (10 nM), K^+^ (1 mM), Na^+^ (1 mM), Ca^2+^ (1 mM), Mg^2+^ (1 mM), Fe^3+^ (1 mM) and Glu (1 mM).

To evaluate the reproducibility of the STX aptasensor fabrication process, five independent batches of sensors were prepared under identical conditions, with three electrodes in each batch. All sensors were incubated with 100 nM of STX at 25 °C for 60 min, followed by DPV measurement. As shown in Figure 6a,b, the peak currents of all electrodes ranged from 6.75 to 7.31 μA, with an average value of 7.05 μA and an RSD below 5%. The between-batch standard deviation was less than 0.13 μA. These results indicate good fabrication reproducibility of the AuNP/CS modification and MB-Apt functionalization procedure.

The storage stability of the STX aptasensor was further evaluated by storing the sensors in a desiccator at 4 °C in the dark. DPV measurements were performed every 5 days under the same assay conditions (100 nM of STX, 25 °C and 60 min; n = 3). As shown in Figure 6c, the current response decreased by only approximately 10% over 25 days, with an RSD below 5%. This result demonstrates that the fabricated aptasensor maintained good long-term storage stability.

The selectivity of the STX aptasensor was examined using a structurally related analogue and common coexisting ions. The sensor responses toward STX, neoSTX and a non-binding mutant (NBM, TGTTCCAGTGAAAGAGCAACAGCAGCAG) were tested at the same concentration of 10 nM in PBS, whereas K^+^, Na^+^, Ca^2+^, Mg^2+^, Fe^3+^ and Glucose (Glu) were tested at 1 mM (Figure 6d). STX produced a pronounced current response, with a ΔI of approximately 1.55 μA, which was defined as a 100% relative response. The natural analogue neoSTX produced a moderate cross-response of approximately 45% (ΔI ≈ 0.70 μA), probably because it shares a similar guanidinium-rich scaffold with STX and can partially interact with the STX-binding aptamer. However, its response remained substantially lower than that of STX. In contrast, the responses induced by K^+^, Na^+^, Ca^2+^, Mg^2+^ and Fe^3+^ were negligible, with ΔI values of 0.12–0.20 μA and relative responses below 15%, close to the blank level. These results show that the aptasensor preferentially responded to STX over the tested analogue and common ionic interferents, supporting its potential application in complex aqueous matrices. The selective validation of other similar toxins has been performed in previous studies [43,44].

**Figure 7:**
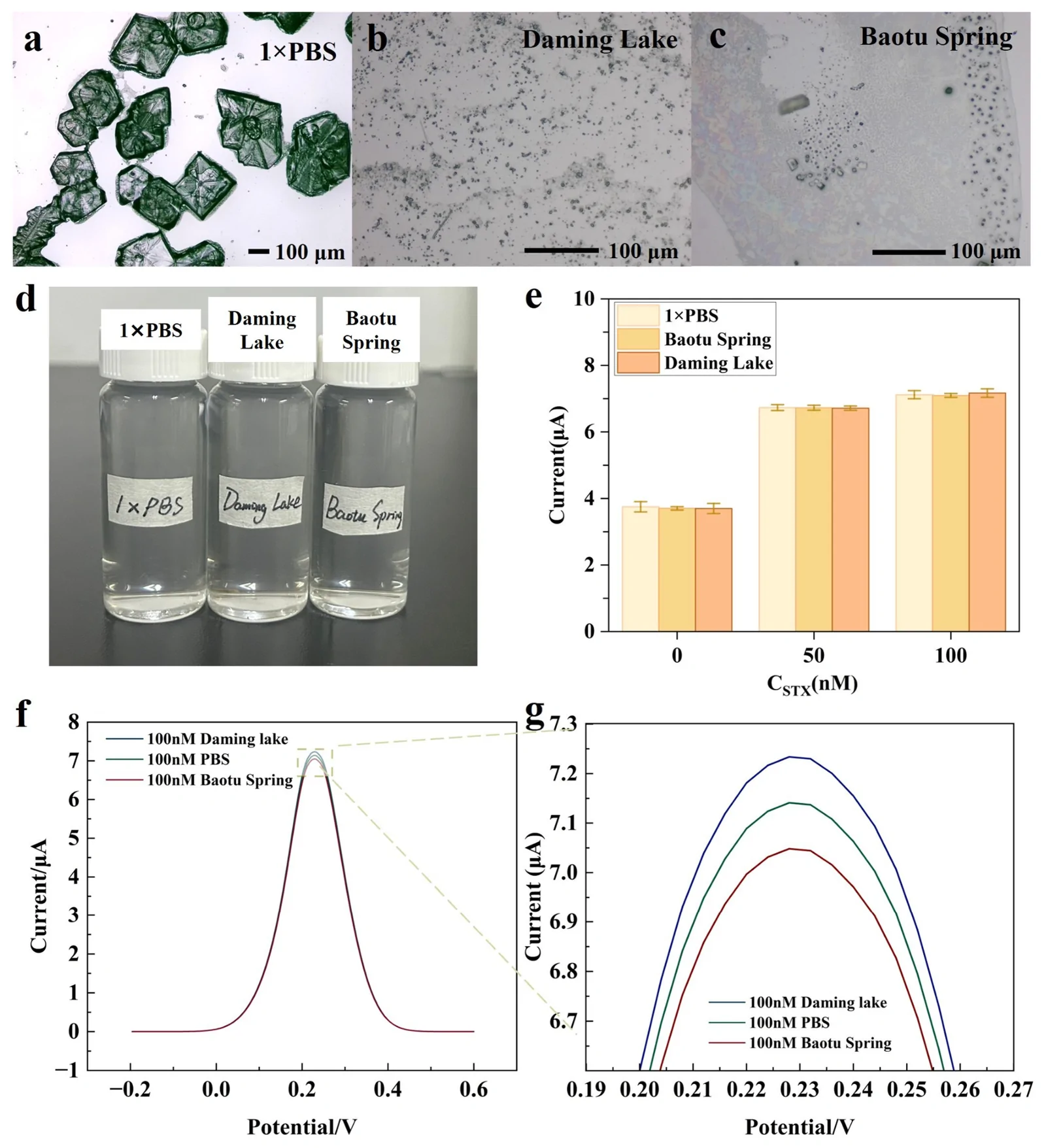
Microscopic images of real water samples for testing freshwater samples. (**a**) 1xPBS, (**b**) Daming Lake, (**c**) Baotu Spring, (**d**) water samples, and (**e**) STX simulation detection in real freshwater samples, with current reactions of adding 0, 50, and 100 nM STX to three matrices. The error bar represents the standard deviation of three repeated measurements. (**f**) DPV curves of 100 nM of STX in PBS, Baotou Spring freshwater samples, and Daming Lake freshwater samples. (**g**) Enlarged image of peak current region.

To assess the applicability of the STX aptasensor in real water matrices, water samples collected from Baotu Spring and Daming Lake were used for spike-and-recovery tests (Figure 7a–d). PBS, Baotu Spring freshwater samples and Daming Lake freshwater samples were spiked with STX at 0, 50 and 100 nM to simulate contaminated freshwater samples. The spiked samples were then analyzed by DPV using the fabricated STX aptasensor.

At 0 nM of STX, the peak current was approximately 3.7 μA, which was used as the background response of the matrix. After STX addition, the DPV peak current increased with an increase in spiked concentration from 50 to 100 nM (Figure 7e). The DPV responses recorded at approximately 0.23 V were comparable among PBS, Baotu Spring and Daming Lake freshwater samples, with peak-current deviations below 5% (Figure 7f,g), indicating limited matrix interference under the tested conditions.

The measured STX concentrations were calculated by substituting the peak-current responses into the calibration equation: y = 1.43log10x + 4.24. Spike recovery was calculated as follows: Recovery (%) = Cmeasured − C_0_/Cjoined × 100%, where Cmeasured is the measured STX concentration, C_0_ is not joined STX, and Cjoined is the added STX concentration. The actual measured concentrations of the spiked samples corresponding to concentrations of 0, 50, and 100 nM are shown in Table S1. Across the tested water matrices, the recoveries ranged from 99.10% to 111.33% (n = 3). These results demonstrate that the aptasensor can detect STX in real freshwater samples and has potential for application in environmental water monitoring.

## 4. Discussion

This study developed a AuNP/CS-based electrochemical aptasensor for STX detection in freshwater samples. The main advantage of this platform is the integration of a chitosan-supported AuNP conductive network with a methylene-blue-labeled STX aptamer on a screen-printed carbon electrode. Chitosan provided a stable scaffold for probe immobilization, whereas AuNPs supported Au–S anchoring and promoted interfacial electron transfer. This architecture enabled signal-on DPV detection of STX with a linear range of 1–1000 nM and a limit of detection of 0.74 nM. Compared with previously reported STX electrochemical aptasensors, this study has a wider detection range while maintaining a low detection limit (Table 1). The electrochemical and simulation results support the role of the AuNP/CS interface in improving sensor performance. COMSOL simulation indicated that interconnected AuNP pathways reduced the equivalent resistance of the composite, while CV and EIS measurements showed an increased electroactive surface area and reduced charge-transfer resistance after modification. The sensor also showed good operational robustness, including room-temperature detection, batch-to-batch reproducibility with an RSD below 5%, and retention of approximately 93% of its initial response after 25 days of storage at 4 °C.

Spike and recovery tests in Baotu Spring and Daming Lake water further demonstrated practical applicability, with recoveries of 99.10%–111.33% and limited matrix interference. However, several limitations remain. neoSTX produced a moderate cross-response of approximately 45%, indicating that the aptasensor may not fully distinguish STX from structurally related analogues. In addition, validation was limited to filtered, spiked freshwater samples under controlled laboratory conditions. Future work should evaluate naturally contaminated samples, broader toxin analogues and field use conditions to support practical deployment for on-site STX monitoring. Although M-30f has been previously validated with a reported K_d_ of 133 nM [42], this study did not perform a construct-matched solution-phase K_d_-gate assay for the MB-labeled and surface-immobilized M-30f under our exact sensing conditions. Future work should include ITC or ThT fluorescence and non-binding mutants to further validate the M-30f/STX interaction.

## 5. Conclusions

In this study, we developed an electrochemical aptasensor for STX detection by integrating a methylene-blue-labeled STX aptamer with a AuNP/chitosan conductive network on a screen-printed carbon electrode. The chitosan matrix provided a three-dimensional scaffold for probe immobilization, whereas the interconnected AuNPs promoted interfacial charge transfer and increased the electrochemically accessible surface. COMSOL simulation, SEM/EDX characterization, CV and EIS measurements collectively supported the formation of a conductive AuNP/CS interface with improved electron-transfer properties. Under optimized assay conditions, the aptasensor produced a concentration-dependent signal-on DPV response to STX, with a linear working range of 1–1000 nM and a limit of detection of 0.74 nM, with an apparent dissociation constant K_d_ = 70.29 ± 29.2 nM. The sensor also showed good fabrication reproducibility, with an RSD below 5% across independently prepared electrodes, and retained stable performance during storage at 4 degrees C, with only an approximately 7% decrease in response over 25 days. Selectivity tests indicated a preferential response to STX over common ionic interferents, although the structurally related analogue neoSTX produced a moderate cross-response. In spiked Baotu Spring and Daming Lake freshwater samples, recoveries of 99.10–111.33% and peak-current deviations below 5% confirmed that the platform can operate reliably in representative freshwater matrices. These results demonstrate that the AuNP/CS-based aptasensor provides a simple, sensitive and reproducible strategy for STX monitoring in aquatic environments, while further validation with broader toxin analogues and field samples will be important for practical deployment.

## Figures and Tables

**Table 1:** Comparison of analytical performance of different sensors for STX detection.

Sensor/Platform	Method	Linear Range	LOD	Reference
AuNP/CS conductive network-based aptasensor	DPV	1–1000 nM	0.74 nM	This work
Ti_3_C_2_T_x_ MXene-based aptasensor	CV and EIS	1.0–200 nM	0.03 nM	[31]
PAH-modified EIS aptasensor	EIS	0.5–100 nM	0.05 nM	[32]
K_3_Fe(CN)_6_-regulated AgNP aptasensor	DPV	40–150 nM	1 nM	[30]
CNT/SAM-modified Au electrode aptasensor with MB probe	DPV	0.9–30 nM	0.38 nM	[33]
MB-labeled aptamer on Au electrode	DPV	1–1000 nM	1 nM	[34]

STX: saxitoxin; LOD: limit of detection; AuNP/CS: gold nanoparticle/chitosan; PAH: poly(allylamine hydrochloride); EIS: electrochemical impedance spectroscopy; CNT: carbon nanotube; SAM: self-assembled monolayer; MB: methylene blue; DPV: differential pulse voltammetry; CV: cyclic voltammetry.

## Data Availability

The data are contained within the article.

## Supplementary Materials

The following supporting information can be downloaded at: https://www.mdpi.com/article/10.3390/bios16090489/s1. Figure S1. COMSOL simulation of low concentration AuNP/CS (a) surface conductivity (b) cross-sectional conductivity; Figure S2. SEM of electrode surface modified with AuNP/CS, Scale bar, 1 μm; Figure S3. STX electrode surface binding experiment. (a) The background current response of the sensor with and without modified MB-Apt in PBS solution with 0 nM STX. (b) Current response of unmodified MB-Apt sensor at different concentrations of STX; Figure S4. Modify the current response of STX aptasensor with different concentrations in a 100 nM STX concentration (n = 3); Table S1. Actual measurement peak current, concentration, and spiked recovery rate of spiked samples at concentrations of 0, 50, and 100 nM.
