# Supplementary material for: A Robust Electrochemical Aptasensor Based on a AuNP/Chitosan Conductive Network for Saxitoxin Detection in Freshwater Samples
Source: Biosensors (Basel). 2026 Sep 3;16(9):489. doi: 10.3390/bios16090489 (PMC13604632; doi:10.3390/bios16090489)
Supplement: Supplementary file 1 [file biosensors-16-00489-s001.zip › biosensors-4482360-supplementary.pdf]

Supplementary Figs and Tables

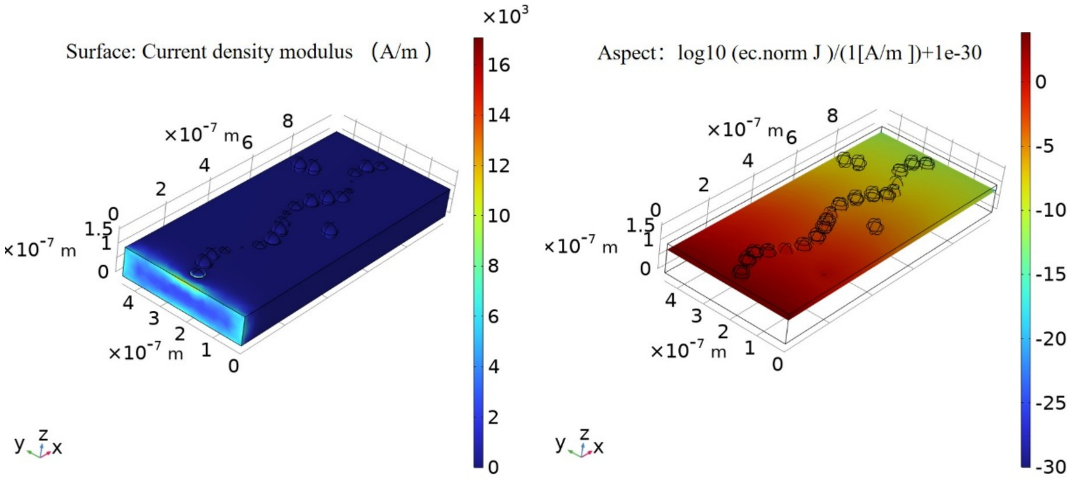

Figure S1. COMSOL simulation of low concentration AuNP/CS (a) surface conductivity (b) cross-sectional conductivity.

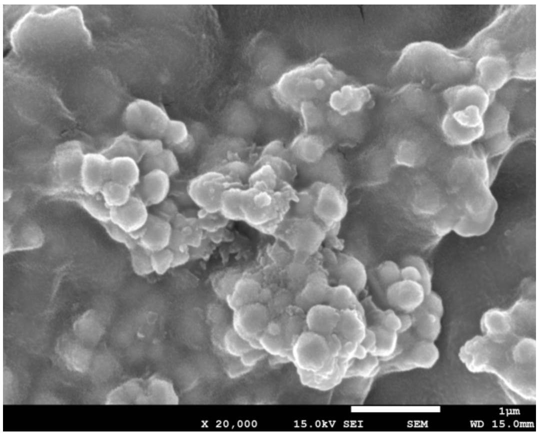

Figure S2. SEM of electrode surface modified with AuNP/CS, Scale bar, 1 μm.

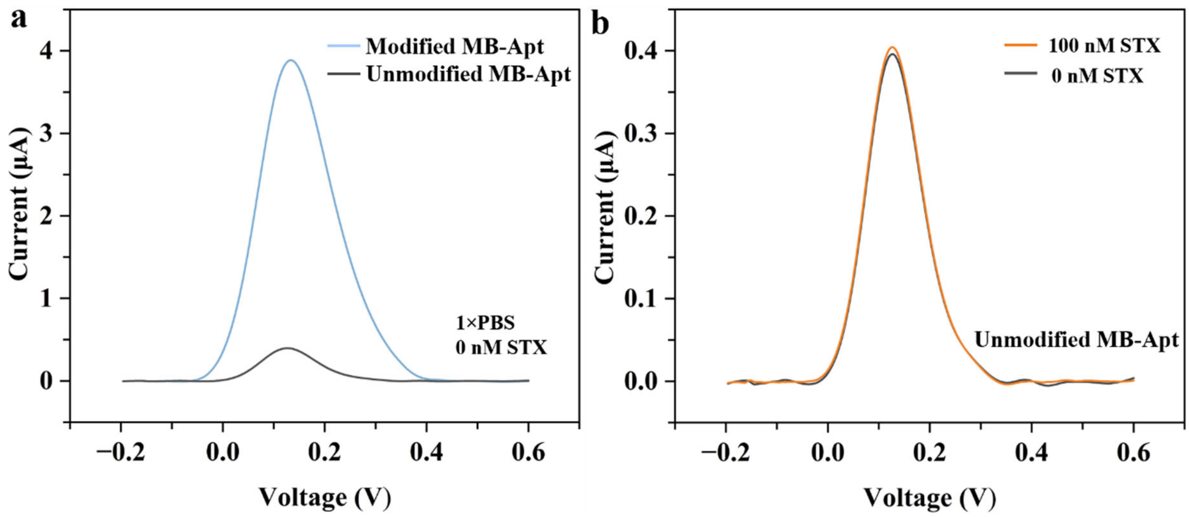

Figure S3. STX electrode surface binding experiment. (a) The background current response of the sensor with and without modified MB-Apt in PBS solution with 0 nM STX. (b) Current response of unmodified MB-Apt sensor at different concentrations of STX.

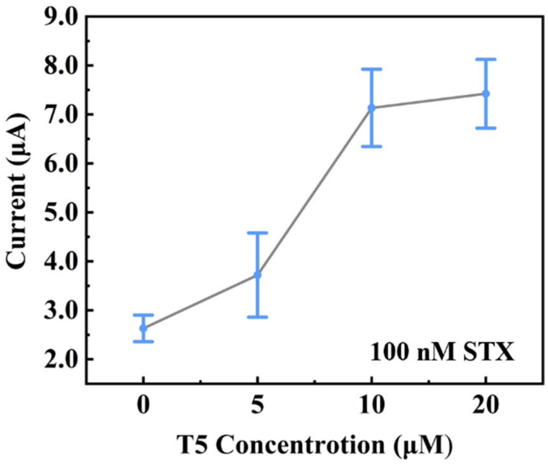

Figure S4. Modify the current response of STX aptasensor with different concentrations in a 100 nM STX concentration (n = 3).

Table S1. Actual measurement peak current, concentration, and spiked recovery rate of spiked sam-ples at concentrations of 0, 50, and 100 nM.

Number	Source	Average Peak current (μA)	C_{joined} (nM)	$C_{measured}$ (nM)	Recovery
1	1×PBS	3.75	0	0.45	—
2		6.73133	50	55.23	109.55
3		7.11933	100	103.16	102.71
4	Baotu Spring	3.70233	0	0.42	—
5		6.727	50	54.85	108.85
6		7.097	100	99.52	99.10
7	Daming Lake	3.69767	0	0.42	—
8		6.71533	50	53.83	106.82
9		7.169	100	111.75	111.33
